# Tips, Tricks, and Potential Pitfalls of CRISPR Genome Editing in *Saccharomyces cerevisiae*

**DOI:** 10.3389/fbioe.2022.924914

**Published:** 2022-05-30

**Authors:** Jacob S. Antony, John M. Hinz, John J. Wyrick

**Affiliations:** ^1^ School of Molecular Biosciences, Washington State University, Pullman, WA, United States; ^2^ Center for Reproductive Biology, Washington State University, Pullman, WA, United States

**Keywords:** Cas9, guide RNA, yeast, synthetic biology, biotechnology, genome engineering, background mutagenesis, off-target mutagenesis

## Abstract

The versatility of clustered regularly interspaced short palindromic repeat (CRISPR)-associated (Cas) genome editing makes it a popular tool for many research and biotechnology applications. Recent advancements in genome editing in eukaryotic organisms, like fungi, allow for precise manipulation of genetic information and fine-tuned control of gene expression. Here, we provide an overview of CRISPR genome editing technologies in yeast, with a particular focus on *Saccharomyces cerevisiae*. We describe the tools and methods that have been previously developed for genome editing in *Saccharomyces cerevisiae* and discuss tips and experimental tricks for promoting efficient, marker-free genome editing in this model organism. These include sgRNA design and expression, multiplexing genome editing, optimizing Cas9 expression, allele-specific editing in diploid cells, and understanding the impact of chromatin on genome editing. Finally, we summarize recent studies describing the potential pitfalls of using CRISPR genome targeting in yeast, including the induction of background mutations.

## Introduction

Clustered regularly interspaced short palindromic repeats (CRISPR)-associated (Cas) systems function as an adaptive immune system against foreign nucleic acids in archaea and bacteria (Marraffini and Sontheimer, 2010; Rath et al., 2015; Jiang and Doudna, 2017; Koonin et al., 2017; Thompson et al., 2021). Using a dual RNA-guided CRISPR endonuclease, such a *Streptococcus pyogenes* Cas9 (spCas9), prokaryotic organisms can specifically recognize and cleave invading foreign DNA (Jiang and Doudna, 2017; Thompson et al., 2021). Crucially, the ability of Cas proteins to target, bind, and cleave selected nucleic acid sequences has been exploited for precise genome editing of eukaryotic organisms (Jinek et al., 2012; Jiang and Doudna, 2017; Thompson et al., 2021). Hence, the development of CRISPR/Cas9 allowed for the rapid expansion of genome engineering into basic research (Giersch and Finnigan, 2017; Thompson et al., 2021) as well as industrial biotechnology and synthetic biology (Stovicek et al., 2015; Raschmanová et al., 2018; Mitsui et al., 2019; Zhang S. et al., 2020; Ding et al., 2020; Malcı et al., 2020; Meng et al., 2020; Molina-Espeja, 2020; Parapouli et al., 2020; Rainha et al., 2020; Patra et al., 2021). Some important applications of CRISPR/Cas9 genome editing applications in *S. cerevisiae* involve the production of biopharmaceuticals, biocatalysts, food additives, chemicals, and biofuels (Hong and Nielsen, 2012; Mattanovich et al., 2014; Auxillos et al., 2019; Mitsui et al., 2019; Kim et al., 2020; Lacerda et al., 2020; Molina-Espeja, 2020; Parapouli et al., 2020; Utomo et al., 2021). Moreover, the expanding CRISPR/Cas toolkit, which includes base editing (Eid et al., 2018; Rees and Liu, 2018; Yang et al., 2019; Anzalone et al., 2020), gene repression and activation (Gilbert et al., 2013; Qi et al., 2013; Didovyk et al., 2016; Dominguez et al., 2016; Brezgin et al., 2019; Pickar-Oliver and Gersbach, 2019; Xu and Qi, 2019; Shakirova et al., 2020) as well as alternative Cas proteins and Cas9 variants (Bao et al., 2015; Nakade et al., 2017; Yan et al., 2019; Paul and Montoya, 2020; Thompson et al., 2021) have begun to greatly expand what can be accomplished with CRISPR/Cas systems in eukaryotic fungi. Our review broadly focuses on the application of various CRISPR technologies for manipulating the genome of *Saccharomyces cerevisiae*. The bulk of this review article addresses how genome editing functions in yeast and describes various experimental tips and tricks for efficient editing, as well as how to avoid potential mutagenic pitfalls associated with CRISPR targeting.

## Pre-CRISPR Marker-Free Genome Editing in *S. cerevisiae*

*Saccharomyces cerevisiae* is a single celled eukaryote that can be stably propagated in either a haploid or diploid state (Jurica and Stoddard, 1999; Duina et al., 2014; Bai et al., 2022)*.* Importantly, *S. cerevisiae* was one of the first eukaryotic species to be genetically modified using transformation methods (Hinnen et al., 1978) and is still a widely used model system for genetic engineering. Due to very efficient homologous recombination pathways in yeast, transformed DNA (*e.g*., PCR products or plasmids) can be readily incorporated into the yeast genome to induce gene deletions, modifications, or insertions of foreign transgenes (Gardner and Jaspersen, 2014). However, these genetic modifications typically require the inclusion of a transgenic selectable marker (*i.e.*, a biosynthetic gene, such as *URA3* or *LEU2* or gene involved in antibiotic resistance) to facilitate selection of rare recombinant cells. The requirement for co-transformation of a marker gene has limited the overall number and types of genetic modifications that can be introduced into a single yeast genome. For example, introducing single nucleotide substitutions in either the promoter or coding region of a yeast gene is challenging using marker-based DNA transformation and recombination. One technique used to perform marker-free genome editing in yeast is the *delitto perfetto* method (Storici et al., 2001; Storici and Resnick, 2006; Stuckey and Storici, 2013). This method involves the integration of a counter selectable reporter (CORE) cassette into a selected genomic location followed by transformation with integrative recombinant oligonucleotides to recombine with the CORE cassette, thereby removing the selectable reporter and introducing the intended genetic modification (Storici et al., 2001; Storici and Resnick, 2006; Stuckey and Storici, 2013). One crucial limitation for this and other alternative techniques is that they typically require multiple steps to construct marker-free genome edits.

The efficiency of genome editing through homologous recombination can be significantly enhanced by introducing a targeted DNA double strand break (DSB) near the intended edit site in the genome. The formation of a DSB induces DNA repair pathways to fix the break, which include homologous recombination pathways utilized in genome editing. In yeast and other eukaryotic species, targeted DSBs have been accomplished using 1) sequence specific endonucleases such as HO or I-SceI (Solis-Escalante et al., 2014; Guha and Edgell, 2017; Fraczek et al., 2018; Gallagher and Haber, 2018), 2) recombinant zinc finger nucleases (ZFN) (Gaj et al., 2013; Peng et al., 2014; Gaj et al., 2016; Chandrasegaran and Carroll, 2016; Guha and Edgell, 2017; Yang and Blenner, 2020), or 3) transcription activator-like effector nucleases (TALENs) (Gaj et al., 2013; Peng et al., 2014; Chandrasegaran and Carroll, 2016; Gaj et al., 2016; Guha and Edgell, 2017), for genome editing purposes (Table 1). However, these technologies are limited due to the difficult and time-consuming steps involved in designing and constructing arrays of ZFN or TALENs that target specific genome sites.

**TABLE 1:** Overview of Genome Editing Technologies.

Pre-CRISPR technologies			CRISPR/Cas technologies		
—	Meganuclease	TALEN or ZFN	Active cas9	Base editing	CRISPRi/a
Editing Outcome	Gene Deletion or Insertion	Gene Deletion or Insertion	Gene Deletion/Insertion/Base Substitution	Base substitution	Reversible gene repression or activation
DNA Repair Events	HR or NHEJ	HR or NHEJ	HR or NHEJ	BER or MMR	N/A
Editing Reagents	I-SceI, HO endonuclease	TALEN (or ZFN) + FokI^a^	Cas9 + sgRNA	dCas9/nCas9 + Deaminase + sgRNA	dCas9 + sgRNA/scRNA + effector(s)
Targeting Mechanism	Binding domain of nuclease	DBD^b^	sgRNA	sgRNA	sgRNA
Multiplex Potential	High	Low, but possible in yeast	High	High	High

a FokI is a restriction enzyme that is fused to arrays of TALENs or ZFNs.

b DNA binding domain.

## Marker-free Genome Editing With CRISPR/Cas9

**FIGURE 1:**
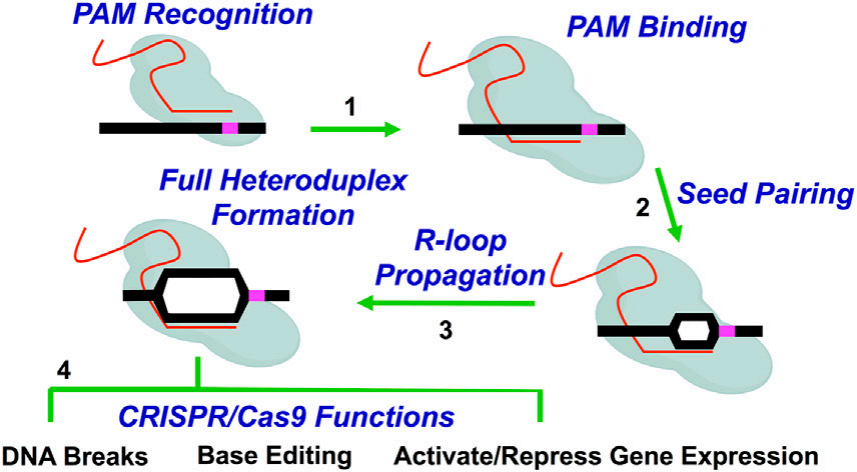
*Overview of Cas9 (or dCas9) targeting mechanism*. Cas9 (or dCas9) recognizes and binds DNA using sgRNA. Briefly, Cas9:sgRNA complexes allow the PAM-interacting domain in Cas9 (or dCas9) to sample PAM motifs in the genome (Step 1). The Cas9:sgRNA complex also aligns the sgRNA for invasion of the target DNA. Following PAM sampling, recognition, and subsequent binding, the DNA duplex is locally unwound and sgRNA begins pairing with complementary target DNA (Step 2). Base pairing in the seed region (*i.e*., the nucleobases immediately adjacent to the PAM motif) is followed by the unidirectional propagation of the DNA:sgRNA heteroduplex (*i.e.*, R-loop) away from the PAM motif (Step 3). Propagation and the subsequent formation of a stable Cas9-induced R-loop is coupled with conformational changes in Cas9 which dictate DNA cleavage activity by the Cas9 endonuclease. Completely paired R-loops for active Cas9 (*i.e*., 20 bp) result in double strand breaks (DSB) 3–4bp from the PAM motif (*i.e.*, Full heteroduplex). The subsequent repair of these Cas9-induced DNA breaks results in the typical mutations observed during genome editing. Alternatively, Cas9 proteins that are involved in base editing or transcriptional modulation will result in deamination activity or gene activation or repression, respectively (Step 4).

CRISPR systems, such as the RNA-guided Cas9 endonuclease from *Streptococcus pyogenes,* have revolutionized genome editing in cells due to the ease of targeting a specific DNA DSB (Jiang and Doudna, 2017). Unlike the more laborious challenges involved in designing ZFN or TALEN proteins to recognize a specific genomic site, Cas9 can be targeted with relatively high specificity to a genomic target site by designing and constructing a 20 nucleotide (nt) segment of the Cas9-bound single guide RNA (sgRNA) that is complementary to the genomic target site (Jinek et al., 2012) (Figure 1). Additionally, the target site must contain a proto-spacer adjacent motif (PAM) immediately adjacent to the 20 nt target site. For *Streptococcus pyogenes* Cas9, this PAM motif is 5’-NGG-3’; however, 5’-NAG-3’ motifs may also occasionally be recognized (Thompson et al., 2021) (Table 2). Since there are many potential PAM motifs within a genome, Cas9:sgRNA complexes can only spend a short amount of time (<30 ms) at any given PAM motif (Jones et al., 2017). During this time, Cas9:sgRNA complexes must engage PAM sites on linear DNA in an open conformation prior to bending the DNA to locally expose the PAM adjacent nucleotides for interrogation in a closed conformation (Cofsky et al., 2022). This conformational change upon binding at a PAM motif can help to stabilize the phosphate lock loop (Lys1107-Ser1109 in Cas9) which likely licenses sgRNA for invasion into duplex DNA and subsequent R-loop propagation (Cofsky et al., 2022). Importantly, this highlights the crucial function of PAM sequences for Cas9 targeting and explains why mutations in the PAM sites eliminate Cas9 targeting (Jinek et al., 2012). Furthermore, this PAM search and binding mechanism can help to explain why mismatches or mutations in the seed region, (*i.e*., the nucleobases immediately adjacent to the PAM motif) are typically less tolerated than mismatches or mutations in more distal regions of the protospacer (*i.e*., the specifically targeted DNA region in the genome) (Sternberg et al., 2014; Szczelkun et al., 2014; Boyle et al., 2017; Alkan et al., 2018; Klein et al., 2018; Zeng et al., 2018; Zhang L. et al., 2020; Ivanov et al., 2020).

**TABLE 2:** Comparison of Different Cas9 and Cas12a Genome Editing Reagents.

Cas9 enzymes	PAM Motif	Protospacer size
SpCas9	5’-NGG-3’^a^	20 bp
SaCas9	5’-NNGRRT-3’	20 bp
FnCas9	5’-NGG-3’	20 bp
NmCas9	5’-NNAGAAW-3’	24 bp
**Other cas enzymes**	**PAM Motif** ^b^	**Protospacer Size**
AsCas12a	5’-TTTN-3’	23 bp
LbCas12a	5’-TTTN-3’	23 bp
FnCas12a	5’-TTN-3’	18–23 bp

a *SpCas9 can also reportedly target non-canonical 5’-NAG-3’ PAM motifs*.

b Cas12a PAM sequence is also reported as 5’-TTTV-3’ in some cases.

When there is perfect complementarity between the sgRNA and the DNA target site, the target DNA duplex is unwound and sgRNA unidirectionally hybridizes with target DNA (Sternberg et al., 2014; Szczelkun et al., 2014; Zeng et al., 2018) (Figure 1). Propagation of a fully complemented sgRNA:DNA heteroduplex (*i.e.,* R-loop) coincides with extensive conformational changes in the Cas9 protein (*ex*. REC2 lobe, HNH domain, RuvC-like domain) which serve to activate Cas9 catalytic activity and position Cas9 nuclease domains (*ex.* HNH domain and RuvC-like domain) for cleavage of target DNA∼3–5 bp upstream of the PAM motif (Cencic et al., 2014; Sternberg et al., 2015; Dagdas et al., 2017; Jiang and Doudna, 2017; Gong et al., 2018; Sung et al., 2018). This commonly results in a blunt-ended DNA DSB or even non-blunted DSBs, both of which trigger endogenous double strand break DNA repair pathways such as homologous recombination (HR) or non-homologous end joining (NHEJ) within cells (Jiang and Doudna, 2017; Brinkman et al., 2018; Stephenson et al., 2018; Xue and Greene, 2021; Nambiar et al., 2022). Importantly, studies in non-yeast eukaryotic systems have shown DSB repair outcomes are non-random and dependent on cell cycle stage and the sequence of the Cas9 target site (van Overbeek et al., 2016; Allen et al., 2018; Taheri-Ghahfarokhi et al., 2018).

In contrast to mammalian cells and many other eukaryotic species, *S. cerevisiae* primarily repairs Cas9-induced DSBs by HR, because NHEJ is inefficient relative to HR (Giersch and Finnigan, 2017; Fraczek et al., 2018). For this reason, Cas9 is not typically used to generate random insertion/deletions mutations in targeted genes in yeast, as is the case in mammalian cells and other eukaryotic species, since these random mutations are typically the outcome of NHEJ repair (Rodgers and McVey, 2016; Jiang and Doudna, 2017; Hanscom and McVey, 2020; Xue and Greene, 2021; Nambiar et al., 2022). Moreover, it is not uncommon to see low levels of random mutations (presumably from NHEJ) at Cas9 target sites, particularly in the absence of a homologous donor template, but these occur at low frequency and are typically comprised of small deletion or insertion events within the target site, often coinciding with the location of the Cas9-induced DSB (Laughery et al., 2015; Lemos et al., 2018; Laughery and Wyrick, 2019). Additionally, larger deletions or chromosomal rearrangements have also been detected (Maddalo et al., 2014; Hao et al., 2016; Kosicki et al., 2018; Mosbach et al., 2020).

**FIGURE 2:**
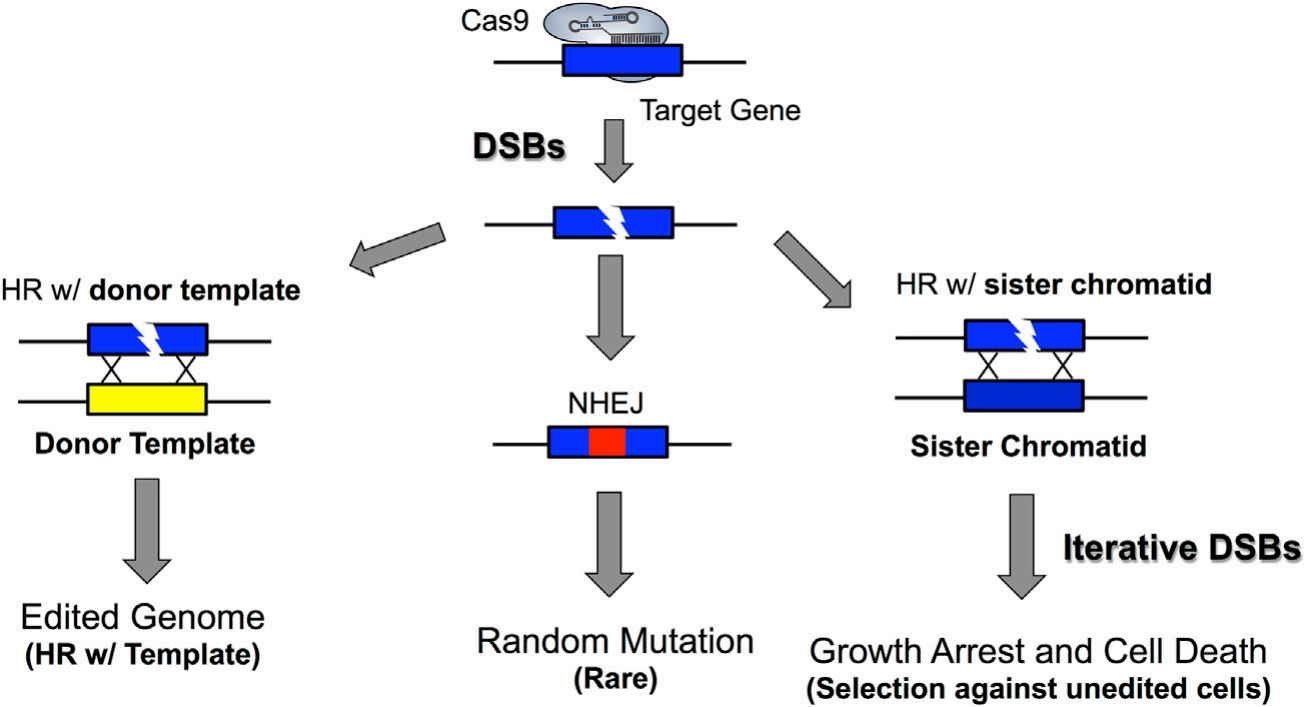
*DSB repair in S. cerevisiae allows for marker-free genome editing.* The high efficiency of homologous recombination in yeast allows homologous donor templates such as oligonucleotides or PCR products to be efficiently incorporated into yeast cells. However, if cells escape Cas9 editing or editing occurs outside the PAM motif or sgRNA targeting space, it is possible that Cas9 could continually target DNA and iteratively form DSBs in the yeast genome. Additionally, this could lead to a low frequency of non-homologous end joining repair of DSBs, which generates random indels at the Cas9 target site. Importantly, the toxicity of Cas9-induced DSBs allows for strong selection against unedited cells because unedited cells typically die, especially if many DSBs are being generated under continual and repeated Cas9 targeting. This highlights a critical parameter for marker-free genome editing in yeast in that desired genomic edits can be obtained by selecting for yeast cells that actively express Cas9 and sgRNA. Moreover, this demonstrates how PAM inactivating mutations, which abrogate CRISPR/Cas9 targeting can be used to minimize the potential detrimental effects associated with excessive and iterative generation of DSBs while also allowing for correct genomic edits to still be enriched for under selective pressure (see key references: Garst et al., 2017; Gorter de Vries et al., 2019; Laughery and Wyrick, 2019).

Cas9 genome editing in yeast typically involves recombination with a homologous donor template that contains the desired genome edit (Figure 2). The design of the homologous donor template involves synthesizing deoxyoligonucleotides that are homologous to the Cas9 target site and contain the desired genome edit. These donor templates can range in size from 90–120+ nucleotides in length and can be either single- or double-stranded DNA. Often, editing efficiency can be somewhat higher with double-stranded DNA donor templates, but single-stranded donor templates are usually sufficient for most genome edits. This donor template is typically co-transformed with Cas9 and sgRNA expressing plasmids to introduce the desired genome edit (DiCarlo et al., 2013; Roy et al., 2018). Moreover, PCR products can also be used as a donor template for marker-free Cas9 genome editing. Often, these PCR products are amplified with primers containing extensive 5’ regions (*e.g.*, 40–50 nt) that are homologous to the integration target in the yeast genome. In this case, it is important to remove all primer dimers that arise during the PCR prior to transformation, typically by gel purification of the full-length PCR product (Laughery and Wyrick, 2019). Removing primer dimers is important because their homology to the genome means they can also serve as a donor template during Cas9-mediated genome editing, and therefore compete with the full-length PCR product during recombinational repair of the Cas9-induced DNA break. This could potentially result in a subset of edited colonies in which the primer dimer is integrated instead of the desired full length PCR product.

Importantly, the donor template must contain one or more base changes that disrupt either the sgRNA target site or the adjacent PAM. This is critical to prevent Cas9 from targeting this location in the genome after the desired genome edit has been introduced by homologous recombination with the donor template (Figure 2). Since even a single base change in the conserved nucleotides of the PAM can eliminate Cas9 targeting, PAM mutations are usually the method of choice to prevent Cas9 from targeting the edited genome (Laughery et al., 2015; Laughery and Wyrick, 2019). However, one or more mutations in the target site complementary to the sgRNA guide, particularly if in the seed region near the PAM, may also prevent Cas9 targeting and result in successful genome editing. Ideally, the same mutation can both introduce the desired genome edit and eliminate subsequent Cas9 targeting (*e.g.,* mutate the PAM) simultaneously. This is often possible when constructing deletion mutations, since frequently the deletion can remove the desired DNA and simultaneously destroy the PAM or target site. However, for many other site-directed mutations, it is necessary to introduce at least two distinct mutations in the donor template, one to inactivate the PAM and the other to introduce the desired genome edit. In this case the two mutations should be relatively close to each other, ideally less than 20 nucleotides apart. Otherwise, it is possible that only one of the mutations (typically the PAM inactivating mutation) will be introduced, instead of both intended edits (Garst et al., 2017; Laughery and Wyrick, 2019).

Unlike the more laborious recombination-based methods used in the past, CRISPR-mediated genome editing in *S. cerevisiae* is relatively simple due to the ease and high efficiency with which yeast can be transformed with Cas9 and sgRNA expression vectors. Therefore, genome editing in yeast can typically be performed without the need to integrate a transgenic marker gene to select for rare recombinants. This marker-free genome editing is possible because all unedited cells are repeatedly subjected to growth-inhibiting DNA DSBs induced by Cas9 at the target site (Figure 2). In contrast, the genomes of edited cells are no longer cleaved by Cas9 because the target site has been disrupted by the genome edit and thus are able to grow (Figure 2). Hence, there is strong selection for the desired genome edit simply by selecting for yeast cells actively expressing Cas9 and sgRNA plasmids, since Cas9 targeting prevents the growth of unedited cells. Importantly, this negative selection is diagnostic of efficient Cas9 targeting and cleavage since transformation of Cas9 and sgRNA plasmids should result in relatively few transformed colonies in the absence a donor template. When donor templates are included in the transformation, many more colonies (*e.g.,* ∼10- to 100-fold more) can be recovered (DiCarlo et al., 2013; Laughery et al., 2015; Laughery and Wyrick, 2019). The few transformed colonies obtained in the absence of the donor template typically have a small deletion (or insertion) event near the Cas9 cut site (Laughery et al., 2015; Laughery and Wyrick, 2019) indicative of either NHEJ repair or unedited cells surviving negative selection by Cas9. Indeed, even colonies arising from donor template-induced genome edits in many cases contain a small fraction of unedited cells (Laughery et al., 2015; Laughery and Wyrick, 2019). Hence, it is important to subject these colonies to a second round of selection for the Cas9 and sgRNA expressing colonies to eliminate these lingering unedited cells.

## Cellular Factors Effecting Cas9 Genome Editing Outcomes

Crucially, CRISPR genome editing in *Saccharomyces cerevisiae* must function within a DNA target site that is packaged into chromatin. The fundamental building block of chromatin is the nucleosome, where ∼147 bp of DNA is wrapped ∼1.67 times around an octamer of histone proteins (Luger et al., 1997; Fierz and Poirier, 2019). Since many DNA enzymes, including DNA methyltransferases, restriction endonucleases, and DNA repair proteins, have greatly reduced activity on nucleosomal DNA substrates both *in vitro* and *in vivo* (Polach and Widom, 1999; Fatemi et al., 2005; Rodriguez et al., 2015; Li and Delaney, 2019; Mao et al., 2020) it was important to characterize how eukaryotic nucleosomes impact the editing efficiency of prokaryotic CRISPR enzymes.

**FIGURE 3:**
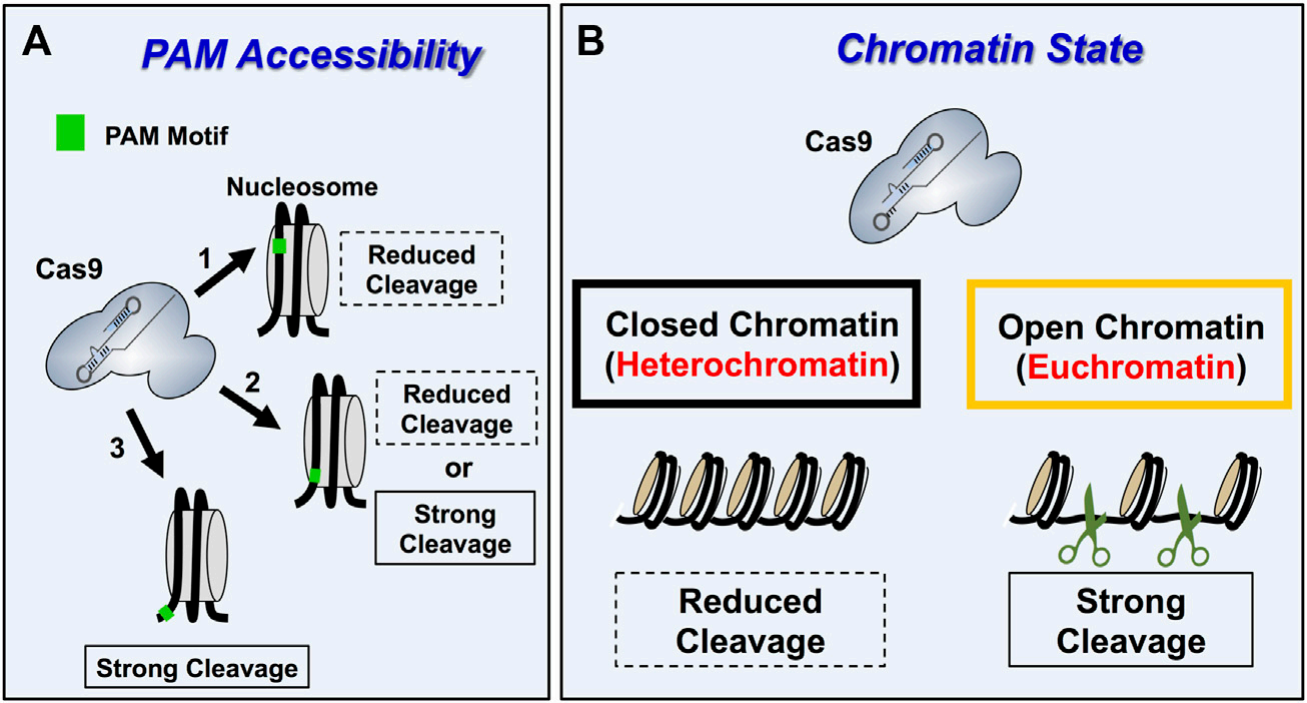
*Overview of Cas9 specificity and activity in eukaryotic chromatin.* **(A)** Schematic describing the impact of PAM accessibility in nucleosome on Cas9 cleavage dynamics. Nucleosomes occlude Cas9 access to PAM motifs within chromatin reducing Cas9 binding and cleavage activity (arrow 1). PAM motifs within linker DNA, which are located outside the nucleosomes making up the chromatin are accessible and thus get cleaved strongly (arrow 3). PAM motifs located at the entry/exit sites of nucleosomes (arrow 2) display variable Cas9 activity because Cas9 activity is influenced by PAM orientation and nucleosome breathing dynamics for these PAM positions. Importantly, PAM motifs oriented away from the nucleosome at the entry/exit sites are typically cleaved better than inward facing PAM motifs. **(B)** Schematic showing how Cas9 targeting (*i.e*., binding and cleavage). is more efficient in open and transcriptionally active euchromatic regions relative to silent, transcriptionally silent heterochromatin regions.

Initial *in vitro* studies indicated that even the packaging of DNA into nucleosomes, which comprises the lowest level of DNA packaging in chromatin, can significantly inhibit Cas9 cleavage of target sites (Hinz et al., 2015; Hinz et al., 2016; Horlbeck et al., 2016; Isaac et al., 2016). The impact of nucleosomes on Cas9 nuclease activity is dependent on multiple factors, including nucleosome positioning strength, location of the PAM motif and target site relative to the nucleosome (Figure 3A), and the presence of ATP-dependent nucleosome remodeling enzymes. For example, target sites in which the PAM is located inside a strongly positioned nucleosome are refractory to Cas9 cleavage *in vitro*. In contrast, PAM sites located in adjacent linker can still be efficiently targeted and cleaved, even if the remainder of the target site is located inside the nucleosome. This suggests that PAM accessibility in chromatin is a critical factor regulating Cas9 targeting and cleavage (Hinz et al., 2015). Moreover, the presence of ATP-dependent nucleosome remodeling enzymes, which can shift nucleosome positions, or intrinsic nucleosomal DNA breathing, particularly near the DNA ends of weakly positioned nucleosomes, can also facilitate Cas9 cleavage of nucleosomal DNA substrates (Horlbeck et al., 2016; Isaac et al., 2016). Interestingly, Cas9 activity at mismatch-containing off-target sites is often more inhibited by nucleosomes than at perfect match on-target sites, suggesting that chromatin may in some cases facilitate Cas9 specificity by suppressing off-target cleavage (Hinz et al., 2016).

There is also accumulating evidence that chromatin impacts Cas9 editing efficiency in cells (Horlbeck et al., 2016; Yarrington et al., 2018). For example, nucleosome-depleted regions in yeast are more readily cleaved by Cas9 endonuclease, and experimentally reducing nucleosome occupancy in nucleosome rich regions of the *HO* and *PHO5* promoters in *S. cerevisiae* enhanced Cas9 cleavage activity (Yarrington et al., 2018). This is consistent with previous studies in other eukaryotic cells indicating that both Cas9 endonuclease activity and catalytically inactive dead Cas9 (dCas9) binding is more inhibited in heterochromatic regions, which are nucleosome rich, relative to euchromatic regions, which are more nucleosome depleted (Kuscu et al., 2014; Wu et al., 2014; Daer et al., 2017; Jensen K. T. et al., 2017; Kallimasioti-Pazi et al., 2018) (Figure 3B). Interestingly, this suggests that euchromatic regions, particularly near promoters, could be targets for off-target mutagenesis by Cas9 (Knight et al., 2015). Anecdotal reports from our own laboratory indicate that marker-free CRISPR editing at yeast telomeres is less efficient than elsewhere in the genome, likely because Cas9 cleavage occurs much less efficiently in this inaccessible heterochromatin context. While the studies above primarily focused on Cas9 activity in chromatin, a recent report indicates that the activity of other CRISPR enzymes (*i.e.,* Cas12a) can also be inhibited by nucleosomes *in vitro* (Strohkendl et al., 2021). Taken together, these findings indicate that chromatin environments in living cells represent an important determinant for target specificity, as well as how successfully an intended genome editing outcome will be achieved.

## Practical Considerations for CRISPR Genome Editing in Yeast

**FIGURE 4:**
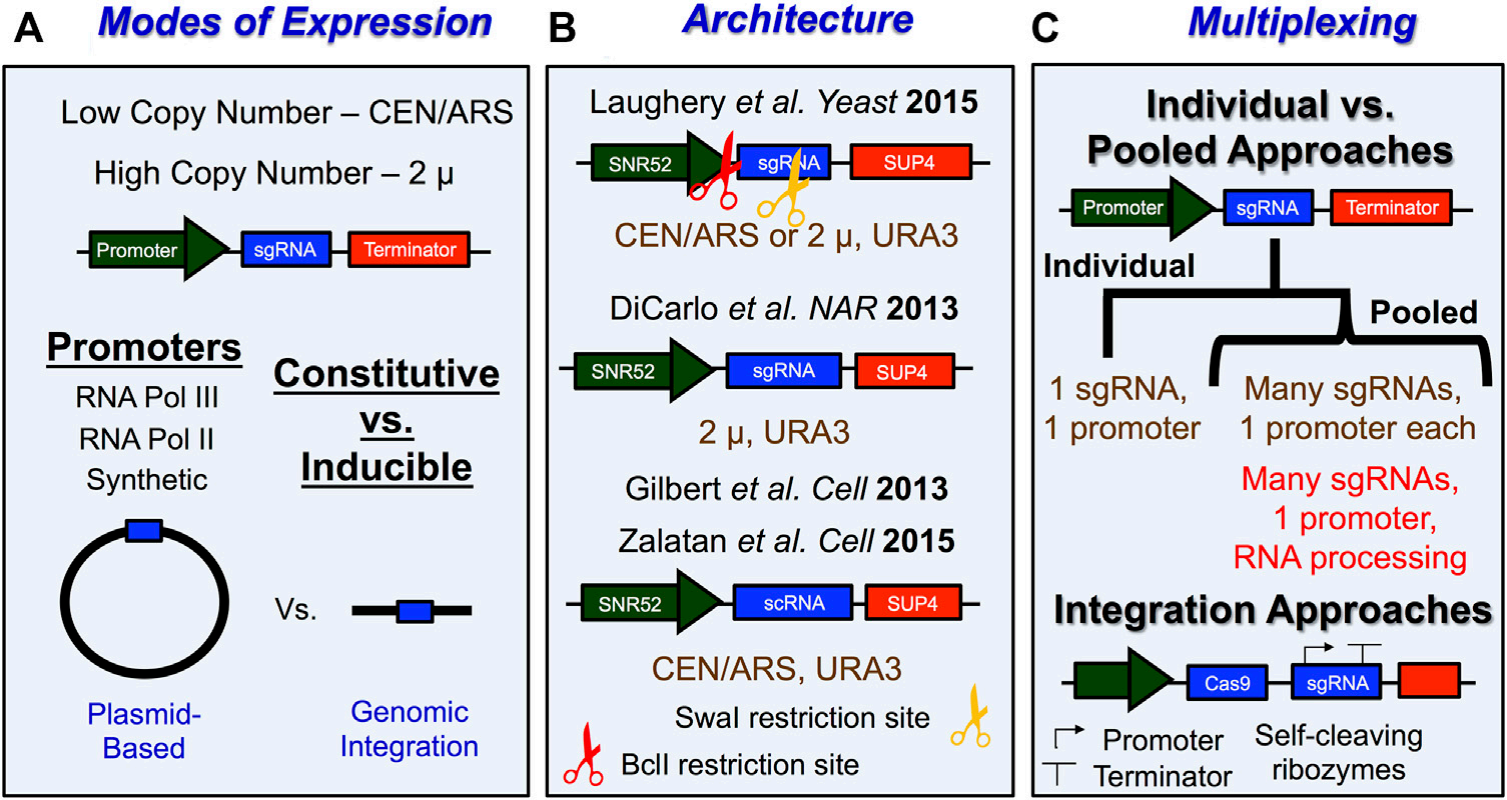
*Tips and tricks for expressing sgRNA in S. cerevisiae.* **(A)** Schematic with overview of important experimental considerations for how sgRNA is expressed in yeast. sgRNA is typically expressed from vectors with low-copy number origins of replication like CEN/ARS or from high copy number origins of replication like 2 µ. Promoters for sgRNA expression typically involve either constitutive or inducible expression of individual or multiple sgRNAs from either a RNA Pol III promoter, RNA Pol II promoter, or a synthetic promoter. Lastly, sgRNAs that get expressed in yeast cells are typically transcribed from plasmid-based vectors or from elements that were directly integrated into the yeast genome. **(B)** Examples of sgRNA expression vector architecture. URA3 is a commonly used selection marker for sgRNA expression vectors and can be used to select for transformants as well as to remove sgRNA expression machinery from yeast cells. LEU2 markers have also been used. BclI (3’ of promoter) and SwaI (in the sgRNA) restriction sites are used for simplifying construction of new targeting sgRNAs in sgRNA vectors. This is done by digesting the plasmid, then hybridizing a user-defined 20 nt DNA segment with a 5’ GATC overhang to facilitate the subsequent ligation of this cassette into the plasmid. **(C)** Schematic describing general approach for multiplexing CRISPR/Cas9 genome editing experiments in yeast. One key consideration is whether a plasmid-based or integration approach is going to be utilized. Plasmid-based approaches will typically involve pooled systems in which many different sgRNAs are expressed from separate promoters within the same construct or the different sgRNAs are generated upon independent processing by ribozymes or other RNA processing enzymes. Strategies employing gRNAs that are flanked by cleavage RNA sequences make use of self-cleavable ribozyme sequences (*e.g*., Hammerhead ribozyme and HDV ribozyme), exogenous cleavage factor recognition sequences (*e.g*., Cys4), and endogenous RNA processing sequences (*e.g*., tRNA sequences and introns). For more details see Stovicek et al., 2017; Lian et al., 2018; Raschmanová et al., 2018; Ding et al., 2020; Malcı et al., 2020; Utomo et al., 2021; Zhang et al., 2021.

**FIGURE 5:**
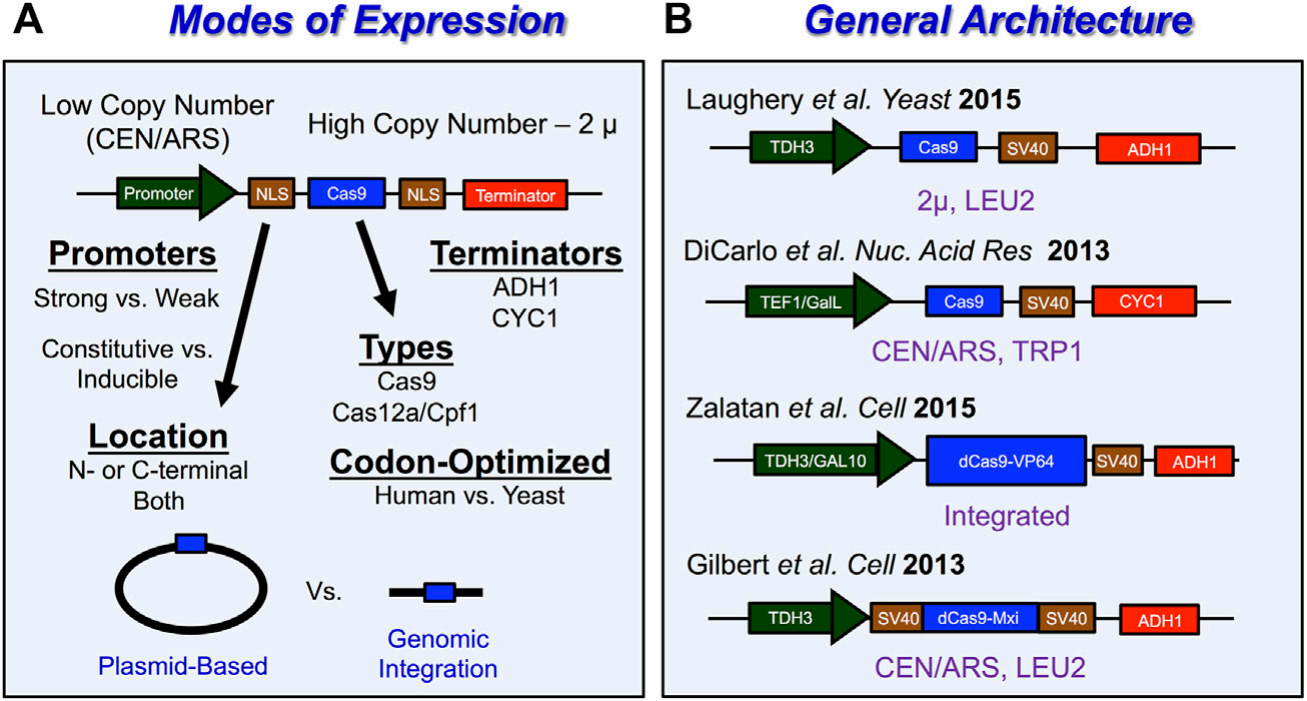
*Tips and tricks for expressing Cas9 in S. cerevisiae* **(A)** Schematic with overview of important experimental considerations for how Cas9 is expressed in yeast. Cas9 is typically expressed from vectors with low-copy number origins of replication like CEN/ARS or from high copy number origins of replication like 2 µ. Promoters for Cas9 expression typically involve either strong or weak promoters. The choice of constitutive or inducible expression and selection of promoter strength for Cas9 are important considerations when looking to avoid potential Cas9-induced toxicity throughout the genome. A nuclear localization signal (NLS) is required to localize Cas9 in the nucleus of eukaryotic cells like *S. cerevisiae*. Importantly, Cas9 expression vectors have been used to introduce both Cas9 enzymes as well as Cas12a/Cpf1 enzymes in *S. cerevisiae*. Another consideration for optimizing Cas9 function in cells is codon optimization. Lastly, Cas9 expression vectors require terminators to stop transcription of Cas9. **(B)** Examples of Cas9 expression vector architecture. LEU2 and TRP1 are a commonly used selection markers for Cas9 expression vectors and can be useful to select for transformants as well as removal of Cas9 machinery from yeast cells. For more details see Stovicek et al., 2017; Raschmanová et al., 2018; Ding et al., 2020.

CRISPR genome editing in yeast typically involves transforming plasmids expressing the active Cas9 gene and a guide RNA (gRNA) along with the donor template (DiCarlo et al., 2013; Roy et al., 2018). Here we describe several practical considerations for performing successful CRISPR/Cas9 genome editing experiments in yeast, including optimizing gRNA design, expression, and delivery as well as expressing Cas9 in *Saccharomyces cerevisiae*. We also provide an overview of several plasmid systems that have been developed to express *Streptococcus pyogenes* Cas9 or other CRISPR enzymes in yeast, along with their corresponding gRNA. Moreover, we discuss how to overcome limitations in Cas9 targeting by using alternative Cas proteins. Typically, genome editing experiments, introduce both gRNA and Cas9 into yeast cells as either low copy (∼1–4 copies per cell) or high copy (∼30–60 copies per cell) plasmids, or through integration into the genome (Karim et al., 2013; Stovicek et al., 2017; Lian et al., 2018) (Figures 4,5).

The most common approach for gRNA design utilizes a chimeric single guide RNA (sgRNA), which fuses the targeting crRNA element with the activating tracrRNA element of the dual gRNA system employed by CRISPR systems in bacteria (Jinek et al., 2012). These sgRNAs allow for the formation of a stable complex with Cas9 endonuclease that is primed for DNA targeting (Jiang et al., 2015). Expression of an sgRNA in yeast typically is achieved by transcription of the sgRNA gene from an RNA polymerase III (Pol III) promoter, since using Pol III avoids transcript cleavage and polyadenylation associated with RNA polymerase II transcription, which would be detrimental to sgRNA function, and because Pol III promoters transcribe small RNAs at high levels (Kabadi et al., 2014). Other methods of sgRNA expression include using a transcript with *cis*-regulatory elements associated with tRNAs along with ribozymes to cleave the transcript at their 5’ end (Ryan and Cate, 2014; Ryan et al., 2014) or a Pol III promoter that is flanked by ribozymes that will cleave both ends of the RNA (Gao and Zhao, 2014). Alternatively, some studies have separately expressed both a crRNA array and a tracrRNA from two separate Pol III promoters (Bao et al., 2015).

The most common promoter and terminator combinations for sgRNA expression in yeast involve the *pSNR52* snoRNA promoter and the yeast tRNA gene *SUP4* terminator (Figure 4B). Interestingly, other studies have shown that different Pol III promoters, such as *pSNR6*, *pSCR1*, and *pRPR1* resulted in lower editing efficiency relative to *pSNR52* (Deaner and Alper, 2019). Therefore, we recommend that, in *S. cerevisiae* sgRNAs are expressed from *pSNR52* promoters. The *SUP4* terminator is essentially a string of T nucleotides (*i.e.,* 5’-TTT​TTT​TGT​TTT​TT-3’), which causes termination of Pol III transcription. This feature of Pol III transcription can be problematic if the guide segment contains multiple thymidine nucleotides, since these can lead to premature termination, and therefore weak expression, of the sgRNA. This is particularly problematic near the 3’ end of the 20 nt guide segment of the sgRNA, since this is immediately adjacent to a structural segment of the sgRNA that contains a long T stretch (*i.e*., 5’-GTTTT … ). This is consistent with the reports of several studies showing that T (or U) nucleotides near the 3’ end of the guide segment of the sgRNA results in poor expression (Wu et al., 2014; Xu et al., 2015) leading to inefficient Cas9 cleavage. Such cases will typically result in a large number of unedited background colonies following transformation with the Cas9 and sgRNA plasmid(s), even in the absence of a donor template, since the negative selection due to repeated Cas9 cleavage of the target site is diminished. This issue can, in part, be remedied by picking larger colonies on the transformation plate, since we have anecdotally found that these colonies are more likely to be edited. However, if the editing efficiency is still too low, redesigning the guide RNA to a more favorable targeting sequence is a practical solution as well (Laughery and Wyrick, 2019). Another option is to use highly expressing RNA polymerase II (Pol II) promoters that are flanked by two ribozyme sequences (a 5’ end hammerhead (HH) and 3’ end hepatitis delta virus (HDV)) to express the sgRNA (Nødvig et al., 2015).

Another important experimental consideration for genome editing experiments in *Saccharomyces cerevisiae* involves how the genome editing protein, *Streptococcus pyogenes* Cas9 endonuclease is expressed in yeast cells. Since Cas9 is expressed within the nucleus of eukaryotic cells, their expression vectors generally require a nuclear localization signal (NLS) such as the SV40 NLS, which is fused to the N- or C-terminus of Cas9 (Figure 5A). Among the other important considerations include whether the *Cas9* gene is optimized to be expressed in yeast, the strength of the promoter expressing Cas9, and the method of expression (low-copy number vs. high copy number plasmid, *etc*.) (Figure 5A). Previous studies have utilized native sequences of Cas9 (Ryan et al., 2014; Bao et al., 2015), human-codon optimized Cas9 (DiCarlo et al., 2013; Gao and Zhao, 2014; Zhang et al., 2014; Jakočiūnas et al., 2015; Laughery et al., 2015; Mans et al., 2015; Stovicek et al., 2015), and even yeast-codon optimized Cas9 (Horwitz et al., 2015; Generoso et al., 2016). Typically, Cas9 is expressed under the control of a strong constitutive promoters from either a low-copy centromeric plasmid (DiCarlo et al., 2013; Gilbert et al., 2013; Zhang et al., 2014; Jakočiūnas et al., 2015; Stovicek et al., 2015; Deaner and Alper, 2019) or a high-copy 2 μ plasmid (Gao and Zhao, 2014; Ryan et al., 2014; Bao et al., 2015; Laughery et al., 2015; Generoso et al., 2016; Shi et al., 2016; Garst et al., 2017; Reider Apel et al., 2017). This plasmid can be either the same as that used to express the sgRNA (*i.e.,* single plasmid system) or a second distinct plasmid with a different selectable marker (*i.e.,* two plasmid system). Some of the most common strong constitutive RNA Polymerase II promoters to express Cas9 include the *pTEF1* promoter (DiCarlo et al., 2013), *pADH3* promoter (Reider Apel et al., 2017), or the *pTDH3* promoter (Gilbert et al., 2013; Laughery et al., 2015; Zalatan et al., 2015; Jensen E. D. et al., 2017) (Figure 5B). Inducible promoters have also been used for Cas9 expression which include the galactose-regulated promoters like *pGalL* (DiCarlo et al., 2013) or *pGAL10* (Zalatan et al., 2015) (Figure 5B). Alternative approaches to Cas9 expression involve integrating Cas9 into the yeast genome (Farzadfard et al., 2013; Horwitz et al., 2015; Zalatan et al., 2015). Regardless of the selected approach to express Cas9 in living yeast cells, there is a possibility that very high levels of Cas9 expression might induce cellular toxicity (Ryan et al., 2014; Generoso et al., 2016). However, the extent to which this occurs may be somewhat dependent on the inherent genetic background of the selected yeast strain, as well as the Cas9 expression method, as other studies have shown that high constitutive expression of Cas9 is generally not cytotoxic (Gao and Zhao, 2014; Bao et al., 2015; Laughery et al., 2015).

Typically, the rate limiting step in many CRISPR editing experiment is cloning the specific 20 nt guide segment in the sgRNA construct to target the genomic region of interest. This 20 nt guide segment is located at the 5’ end of the sgRNA, immediately after the promoter driving guide RNA expression. For this reason, it can be challenging to efficiently introduce the 20 nt guide segment into its proper location in the sgRNA expression construct. A number of different experimental strategies have been developed to facilitate guide segment cloning into the sgRNA expression construct. Our own approach for designing and cloning new 20mer targeting guide sequences involves the use of unique internal restriction enzyme sites (BclI and SwaI) that permit rapid, directional cloning of hybridized oligonucleotides containing a 5’ GATC overhang on one end and a blunt end on the other (Laughery et al., 2015; Laughery and Wyrick, 2019). This allows for rapid and specific integration of a user-defined 20-mer guide sequences into a selected sgRNA expression vector. Moreover, these vectors can be designed to allow for rapid removal of Cas9 machinery following a period of non-selective growth and replica plating (for PML107, which is a Cas9/sgRNA vector with a *LEU2* selectable marker) or by counter-selection on 5-fluoroorotic acid (5-FOA) containing plates (for PML104, which is a Cas9/sgRNA vector with a *URA3* selectable marker) (Laughery et al., 2015; Laughery and Wyrick, 2019).

Other cloning strategies include using Golden gate assembly to introduce guide sequences into an expression cassette (DiCarlo et al., 2013; Bao et al., 2015) or PCR amplification followed by product circularization and uracil-specific excision reactions (USER) to introduce new 20-mer sgRNA sequences into an expression vector (Bitinaite et al., 2007; Jakočiūnas et al., 2015). This second approach is useful because it is amenable to multiplexing experiments with CRISPR/Cas9. Lastly, some groups have directly transformed PCR products to generate gRNA cassettes (Horwitz et al., 2015) and even synthesized and cloned an entire gene block containing sgRNA cassettes to obtain individual guide sequences (Zhang et al., 2014).

Finally, in some cases it can be difficult to identify a guide RNA target near the desired genome edit due to a lack of neighboring PAM motifs. *Streptococcus pyogenes* Cas9 specifically recognizes 5’-NGG-3’ PAM motifs within the genome (Table 2), and this PAM dependency limits the potential genomic targeting space (Jiang and Doudna, 2017). One trick to bypass this limitation is the use of alternative Cas enzymes with different PAM specificities. One important Cas protein that has been employed for genome editing is the Cas12a enzyme (also known as Cpf1), which recognizes a T-rich PAM motif (Nakade et al., 2017; Yan et al., 2019; Paul and Montoya, 2020; Thompson et al., 2021) (Table 2). Genome editing using Cas12a has a number of differences from Cas9 for genome editing in yeast: first, it does not require tracrRNA, therefore their sgRNAs are shorter relative to Cas9; second, it can process multiple crRNAs from a single crRNA array, whereas for Cas9 one must express crRNAs separately or process gRNAs using additional enzymes; third, its PAM motif is located on the 5’ end of the target DNA sequence whereas this is on the 3’ end for Cas9; and finally Cas12a generates staggered or sticky DSBs containing overhangs, often leading to deletions and point mutations (Świat et al., 2017; Zetsche et al., 2017; Li Z.-H. et al., 2018; Verwaal et al., 2018; Ciurkot et al., 2019; Ciurkot et al., 2021). These properties of Cas12a make multiplexing experiments relatively simple relative to those for Cas9 which require multiple or even complex expression constructs. Another approach to dealing with PAM constraints with Cas9 is to use Cas9 from other bacterial species (Okada et al., 2021) (Table 2).

## Multiplex Genome Editing With CRISPR

Instead of editing a single genomic site with CRISPR, it is possible to express multiple sgRNAs to edit multiple genomic sites in a cell simultaneously (Figure 4C). Some multiplexing approaches involve using many different sgRNA expressing plasmids (Horwitz et al., 2015), and while this allows for up to 5 simultaneous genomic edits, it can be cumbersome to select for edits or remove Cas9 machinery. Other methods make use of pooled or arrayed approaches (Figure 4C). For example, one pioneering approach to advance applications of gene regulation in yeast involves the use of *Pseudomonas aeruginosa* Csy4, which is a bacterial RNA processing ribonuclease, to generate multiple gRNA from a single transcript (Ferreira et al., 2018). Interestingly, this approach has also been applied to genome editing experiments in mammalian eukaryotic systems (Nissim et al., 2014). Another pooled approach for multiplexing CRISPR experiments called HI-CRISPR, utilizes a Cas9 variant, iCas9 (D147Y, P411T) and an sgRNA cassette under the control of the *pSNR52* promoter containing four different sgRNAs interspersed by direct repeats to target up to 4 genes simultaneously (Bao et al., 2015). Interestingly this approach also expresses tracrRNA with iCas9 and sgRNA all on the same expression vector. A different group was able to edit up to 3 targets simultaneously in diploid yeast using an sgRNA array flanked by ribozymes, using a method termed multiplex CRISPR (CRISPRm) (Ryan et al., 2014). Several studies have employed self-cleaving sequences from tRNA for multiplex engineering in *S. cerevisiae* (Deaner et al., 2018; Zhang Y. et al., 2019). One group developed a method termed GTR-CRISPR which employed a gRNA-tRNA array (Zhang Y. et al., 2019) while another group developed a method termed CRISPR-Ligation Extension of sgRNA Operons (LEGO), which uses tRNA-sgRNA (TST) operons with an iterative Type IIs digestion/ligation extension to construct and express sgRNA from a larger sgRNA operon (Deaner et al., 2018). Another plasmid-based approach called CRISPR/Cas9 mediated genome Editing (crEdit) which expresses sgRNA arrays from an episomal 2μ-based vector, while also expressing Cas9 from either a constitutive *pTEF1* promoter or inducible *pCUP1* promoter on an ARS/CEN vector. Importantly, the linearized donor plasmids harbor the desired integration sequences flanked by homology arms as well as the desired genomic modification (Ronda et al., 2015).

While all the previous approaches employed plasmid-based approaches to multiplexing experiment, multiplexing experiments can also be performed by integrating Cas9 and/or sgRNA expression machinery into the yeast genome (Malcı et al., 2020; Utomo et al., 2021; Zhang et al., 2021). One group accomplished this by placing pCut-X, a single Cas9 and sgRNA high-copy expression vector, into the yeast genome (Figure 4C). Here, Cas9 is expressed from an *pADH1* promoter and a *CYC1* terminator while the gRNA cassette within pCut-X is expressed from a tyrosine tRNA promoter fused to a self-cleaving, genomic hepatitis delta virus C ribozyme (Reider Apel et al., 2017). Another study developed an integration-based method called Cas9-facilitated multiloci genome engineering (CasEMBLR) to mediate one-step double-strand breaks at single, double, and triple integration sites (Jakočiūnas et al., 2015; Jakočiūnas et al., 2018). Briefly, this method clones between one-three sgRNAs into an sgRNA expressing cassettes for plasmid-based expression and then plasmids expressing gRNA(s) and linear DNA parts are co-transformed into Cas9-expressing *S. cerevisiae* cells for Cas9-facilitated multiloci genomic integration of *in vivo* assembled DNA parts (Jakočiūnas et al., 2015). A different approach for genomic integration-mediated multiplexing termed multiplexed accurate genome editing with short, trackable, integrated cellular barcodes (MAGESTIC) involved the use of an array of sgRNA-donor DNA oligos to integrate genomic barcode (Roy et al., 2018). This approach is advantageous because 1) it eliminates the need for marker selection, 2) there is one barcode per cell, eliminating the reliance on variable copy number plasmids, and 3) recombinase-directed indexing can be used to screen thousands of individual strains.

## Allele-specific Genome Editing in Diploid Yeast

Another potential application for CRISPR/Cas9 genome editing in yeast is in creating allele specific genome edits in diploid or polyploid genomes. Polyploid genomes or those with heterozygosity, a common theme among yeast hybrids as well as some industrial and natural yeast strains, are more challenging to manipulate successfully (Gorter de Vries et al., 2017; EauClaire and Webb, 2019; Gorter de Vries et al., 2019). This challenge is due, in part, to the presence of undamaged homologous chromosomes, which can be used as a template during repair by homologous recombination. One solution to successfully edit diploid yeast is to design allele-specific gRNAs (Sadhu et al., 2016). This approach results in a DSB being induced on a single homologous chromosome while the other homolog is unaffected and is particularly effective when heterozygous loci have different PAM motifs or different 5’ sequences proximal to a PAM motif (Sadhu et al., 2016). However, diploid genome can experience loss of heterozygosity (LOH) when DSBs are induced, which could limit Cas9 editing efficiency and potentially even cause extensive genetic changes (Fleiss et al., 2019; Gorter de Vries et al., 2019). One possibility to mitigate the risk of mutations associated with LOH would be to design gRNAs that eliminate all alleles of a heterozygous loci, even if only one allele is being targeted for modification (Gorter de Vries et al., 2019). Another approach involves the use of PAM mutations to lock the intended mutations into a chromosome. This approach could be used to generate heterozygous chromosomes containing heterozygous insertions or deletions, introduce homozygous mutations in a diploid, or introduce wild type sequences into a diploid strain. One limitation to this approach is that it requires guide sequences that allow for the introduction of silent mutations in the PAM motif (Gorter de Vries et al., 2019).

## Alternative CRISPR-Based Applications in Yeast

The most essential feature of genome editing experiments is the ability to introduce a desired genetic change in a targeted DNA sequence. However, CRISPR/Cas systems can also be used to alter gene expression or associated epigenetic modifications. Here, we will briefly describe some of the alternative tools and technologies within the CRISPR arsenal, including CRISPR-mediated transcriptional activation (CRISPRa), CRISPR-mediated transcriptional interference (CRISPRi), and base editing (Didovyk et al., 2016; Dominguez et al., 2016; Eid et al., 2018; Rees and Liu, 2018; Brezgin et al., 2019; Pickar-Oliver and Gersbach, 2019; Xu and Qi, 2019; Yang et al., 2019; Anzalone et al., 2020). One unifying principal to all these different CRISPR technologies is that they employ sgRNA-directed targeting of a partially or completely inactivated Cas protein (*ex.* Nickase Cas9; nCas9; D10A or H840A or Dead Cas9; dCas9; D10A and H840A) that is fused to effector proteins to manipulate genomic information either through DNA modification, transcriptional regulation, or epigenetic modifications.

DNA binding by an sgRNA bound dCas9 was first shown to serve as a platform for reversibly modulating sequence-specific changes in gene expression in bacterial and mammalian cells (Qi et al., 2013). Furthermore, dCas9 has also been shown to robustly silence gene expression in yeast and other eukaryotic cells (Gilbert et al., 2013). There are two primary mechanisms through which dCas9 can alter the expression of genes: by directly inhibiting RNA polymerase (Qi et al., 2013), or by directing proteins fused to dCas9 to chromatin or promoter regions. This can either lead to the partial loss of expression and function in a process termed CRISPRi or gain of expression and function with CRISPRa. Importantly, under stable expression of both sgRNA and dCas9, CRISPRi provides robust suppression of gene expression. Activation of gene expression with CRISPRa in yeast has typically involved using dCas9 fused to a strong transcriptional activator, often the VP64 transcription activator from the herpes simplex virus (Farzadfard et al., 2013; Mali et al., 2013; Yu and Marchisio, 2021; Zhang and Marchisio, 2022), or a combination of transcriptional activators with the VPR system (Chavez et al., 2015; Yu and Marchisio, 2021; Zhang and Marchisio, 2022). Importantly, these systems provide significant induction of gene expression at both individual and multiple genomic targets. One pioneering approach for CRISPRa involves the use of scaffold RNA (scRNA) which forms a hairpin aptamer domain at the 3’ end of the sgRNA that recruits aptamer-specific proteins to the dCas9-bound target site to alter gene expression to a greater extent than using dCas9-VP64 alone (Zalatan et al., 2015). Furthermore, combinations of scRNAs can be used to simultaneously activate and repress gene expression (Dominguez et al., 2016; Jensen E. D. et al., 2017). One distinctive limitation to this approach is that using too many copies of the aptamers in the scRNA may reduce their expression limiting the efficiency in altering gene expression.

DNA base editors constitute another important advancement for genome editing tools since they can directly install precise point mutations into cellular DNA without the introduction of double strand breaks and their associated byproducts, which can be mutagenic (Rees and Liu, 2018). Base editors are categorized into two classes: adenine base editors (ABEs) which convert A:T base pairs to G:C base pairs using adenine deaminases (Gaudelli et al., 2017) or cytosine base editors that convert C:G base pairs to A:T base pairs (Komor et al., 2016). Base editing applications in *S. cerevisiae* rely on the fusion of cytosine deaminases such as CDA1 or AID to dCas9 or nickase Cas9 (nCas9) to generate targeted base substitutions (*ex*. C to G or C to T) within a base editing window that is ∼15 bp away from the PAM motif on the displaced single stranded non-complementary strand of the dCas9-induced R-loop (Nishida et al., 2016). Among the limitations to base editing include off-target effects (Park and Beal, 2019) and indel formation resulting from base excision repair activity on deaminated bases and the activity of other DNA repair pathways (*ex.* translesion synthesis, TLS; mismatch repair, MMR; or single or double strand break repair) (Komor et al., 2017; Lei et al., 2018; Rees and Liu, 2018; Anzalone et al., 2020; Jiang et al., 2021).

## Potential Pitfalls to Genome Editing in *Saccharomyces cerevisiae*

**FIGURE 6:**
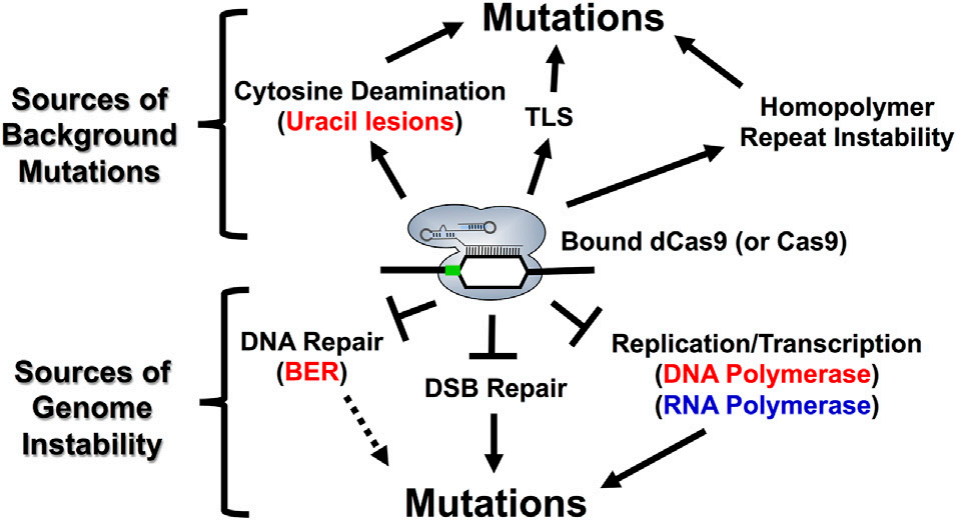
*Model describing the molecular mechanisms associated with Cas9 mutagenesis in S. cerevisiae.* R-loop formation and DNA binding by Cas9 endonuclease can inhibit endogenous cellular processes such as BER, NHEJ, DNA replication and RNA transcription resulting in distinct on- and off-target mutational profiles for both active Cas9 and the dCas9/nCas9 enzymes that are used in either base editing or transcriptional regulation applications. Each of the factors influencing mutagenesis at the top of figure can be generated upon R-loop formation by dCas9. Base substitutions (*ex.* C to T or C to G) can be explained by cytosine deamination and the subsequent activity of BER or TLS enzymes. Complex mutations such as indels and structural variations can be explained, in part, by targeting homopolymer sequences with dCas9 as well as replication stress from bound dCas9 (bottom of figure). Additionally, inhibition of other cellular processes, such as BER, NHEJ, and RNA polymerases are likely to impact both on- and off-target mutational outcomes with Cas9 proteins. For example, the extent to which inhibition of RNA polymerase by Cas9 binding impacts mutagenesis is likely reflected in how readily RNA polymerase can dislodge bound Cas9 from a genomic target site. Notably, RNA polymerase is more likely to dislodge bound Cas9 when it is targeted to the transcribed strand. This likely impacts Cas9 activity in cells by allowing Cas9 to better function as a multi-turnover enzyme *in vivo*. The PAM motif is indicated by the green box. For more details see Gilbert et al., 2013; Clarke et al., 2018; Laughery et al., 2019; Doi et al., 2021; Antony et al., 2022.

An important pitfall in all CRISPR genome editing applications with Cas9 proteins is the possibility of introducing unintended background mutations, often at off-target Cas9 binding sites (Figure 6). One important source of these unwanted outcomes lies in the propensity of Cas9:sgRNA complexes to bind to mismatch-containing off-target sites in the genome, which can result in unanticipated DNA editing events and mutagenesis (Pattanayak et al., 2013; Kuscu et al., 2014; Polstein et al., 2015; Ma et al., 2016; Park and Beal, 2019). Briefly, Cas9 binding kinetics in eukaryotic cells is modulated by the location and number of mismatches between the sgRNA and target DNA, with mismatches near the PAM motif being more detrimental to Cas9 binding and cleavage activity than mismatches more distal from the PAM motif (Boyle et al., 2017; Ivanov et al., 2020). Moreover, off-targeting binding kinetics by Cas9 (or dCas9) can also be impacted by Cas9 expression level, as genome-wide studies in yeast have shown that high expression is associated with more frequent off-target binding relative to lower levels of Cas9 expression (Waldrip et al., 2020). Lastly, incomplete base pairing at off-target binding sites likely locks Cas9 into a cleavage-inactive structural conformation, explaining why Cas9 binds more off-target sites than it cleaves (Kuscu et al., 2014; Wu et al., 2014; Tsai et al., 2015).

Obviously, off-target Cas9 cleavage events can introduce background mutations in yeast cells. However, DNA binding by Cas9, in the absence of DNA cleavage, is also mutagenic in yeast cells (Laughery et al., 2019; Doi et al., 2021). This is important because this indicates that off-target binding as well as off-target cleavage events may contribute to background mutations arising during genome editing experiments. Here, we will highlight some molecular mechanisms that can contribute to unexpected background mutations at Cas9 (or dCas9) binding sites, even in the absence of DNA cleavage.

One mechanism by which Cas9 (or dCas9) binding alone can induce mutations is by inducing spontaneous cytosine deamination in the single-stranded DNA formed in the Cas9 R-loop (Laughery et al., 2019) (Figure 6). This agrees with studies indicating that single-stranded DNA is much more prone to spontaneous cytosine deamination than double stranded DNA (Kim and Jinks-Robertson, 2012; Skourti-Stathaki and Proudfoot, 2014), resulting in more frequent conversion of cytosines to mutagenic uracil lesions. One important finding from this study is that mutagenesis resulting from R-loop formation by dCas9 predominantly induces C to T mutations in an *ung*1-deficient background, which is incapable or removing uracil lesions, at both on and off-target binding sites (Laughery et al., 2019). Interestingly, dCas9 targeting also introduced a high frequency of complex mutation events (*i.e*., multiple nearby single base substitutions and/or insertion/deletion events). These mutations were likely caused by error-prone translesion DNA synthesis (TLS), as *CAN1* mutation frequencies were reduced in a *rev3*-deficient background, which is incapable of undergoing potentially error-prone TLS (Laughery et al., 2019). In summary, these findings indicate that dCas9 (or Cas9) binding can potentially promote background mutations through multiple mechanisms.

Another mechanism in which Cas9 (or dCas9) binding alone can induce mutations is through stalling replicative DNA polymerases, thereby inducing larger structural variations (SVs) in the yeast genome (Doi et al., 2021). Interestingly, this study showed that dCas9 targeting at tandem repeats in arrays of both CUP1, a metallothionein which buffers concentrations of intracellular copper in budding yeast, and *ENA1*, which encodes an ATPase sodium pump, induce copy number expansions or contractions (Doi et al., 2021). Moreover, this study linked the destabilization of tandem repeats to the Rad52-dependent single stranded annealing (SSA) repair pathway, which can rescue collapsed or stalled replication forks (Doi et al., 2021).

Importantly, unwanted background mutations arising from deamination events or repeat instability are dependent on a variety of different DNA repair pathways (Komor et al., 2017; Lei et al., 2018; Laughery et al., 2019; Jiang et al., 2021; Nambiar et al., 2022). The major repair pathways we highlight include base excision repair (BER), mismatch repair (MMR), and translesion synthesis (TLS), which are all functional in *S. cerevisiae* (Boiteux and Jinks-Robertson, 2013; Skoneczna et al., 2015; Mourrain and Boissonneault, 2021). BER is the major pathway for resolving base modifications resulting from deamination, alkylation, or oxidation. Briefly, DNA glycosylases remove the damaged base and AP endonuclease I subsequently nick the DNA backbone generating a single strand break at the site of the base damage. DNA polymerases will then synthesize across this gap and a DNA ligase subsequently seals the newly synthesized DNA to complete BER (Kim and Iii, 2013). MMR recognizes damage arising from replication errors or recombination errors while TLS is involved in bypassing DNA damage through both error-free and error-prone mechanisms (Skoneczna et al., 2015). These repair pathways are more extensively reviewed in the following review articles (Gu et al., 2021; Nambiar et al., 2022). Given the importance of DNA repair for mediating both intended and unwanted mutations with Cas9 (and dCas9), understanding how Cas9 interacts with endogenous DNA repair pathways is becoming increasingly important. A recent *in vitro* study from our group demonstrates that BER enzymes like uracil DNA glycosylase (UDG) are inhibited by dCas9 binding in a position-dependent manner (Antony et al., 2022). This could be important towards understanding both base editing outcomes as well as unanticipated mutations resulting from dCas9 (or Cas9) targeting within cells (Figure 6). For example, inhibition of UDG by dCas9 may help to explain why R-loop formation by dCas9 predominantly induces C to T mutations as well as why some mutations accumulate at specific locations within the dCas9-induced R-loop (Laughery et al., 2019). Moreover, it raises questions about how Cas9 (or dCas9) may interact with other endogenous repair pathways (*ex.* MMR, TLS) which are implicated in determining mutational outcomes resulting from dCas9 binding and base editing.

## Strategies to Reduce Off-Target Binding and Cleavage by Cas Proteins in *S. cerevisiae*.

It is important to carefully design gRNAs to avoid unwanted mutations resulting from off-target binding and cleavage by Cas9. Therefore, we recommend using web-based tools to identify putative gRNA target sites and to assess whether there might be any potentially problematic off-targeting events prior to constructing and optimizing sgRNA expression vectors. Many *in silico* tools have been developed and are extensively reviewed elsewhere (Lee et al., 2016; Stovicek et al., 2017; Raschmanová et al., 2018; Deaner and Alper, 2019; Liu et al., 2019; Ding et al., 2020; Manghwar et al., 2020; Naeem et al., 2020; Sledzinski et al., 2020). A selection of potentially helpful sgRNA design tools for yeast are described in Table 3. Moreover, our laboratory has a gRNA design tool for yeast to identify and display potential gRNA targets from a user-defined target as well as to generate oligonucleotides to construct new sgRNA cassettes (Laughery et al., 2015).

**TABLE 3:** Web-Based Tools for sgRNA Design in S. cerevisiae.

Tools to evaluate sgRNA design			
Name	CRISPR systems	Comments^a^	References(s)
CRISPOR	Cas9, Cas12a	Cas9 alternatives available	Haeussler et al. (2016), Concordet and Haeussler, (2018)
Off-Spotter	Cas9	Other PAM motifs available	Pliatsika and Rigoutsos, (2015)
Cas-OFFinder	Cas9, Cas12a, Cas12b	Cas9 and Cas12a alternatives available	Bae et al. (2014)
CC-Top	Cas9 and Cas12a	Cas9 alternatives available	Stemmer et al. (2015)
CHOPCHOP	Cas9, nCas9, Cas12a	For KO, KI, Repression, or Activation	Montague et al. (2014), Labun et al. (2016), Labun et al. (2019)
CRISPRdirect	Cas9	Other PAM motifs available	Naito et al. (2015)
E-CRISP	Cas9	Other PAM motifs available	Heigwer et al. (2014)
CRISPR-ERA	Cas9, nCas9	For Editing, Repression, or Activation	Liu et al. (2015)
sgRNAcas9	Cas9. nCas9	Can predict off-target cleavage	Xie et al. (2014)

a Knock-out (KO), Knock-In (KI), Cas9 alternatives means SpyCas9 variants or Cas9 from other species, Cas12a alternatives means Cas12a from other species.

Another approach to optimizing the efficiency of genome editing experiments in eukaryotic systems is to limit off-target effects imposed by Cas9 (Jiang et al., 2019; Davidson et al., 2020; Marino et al., 2020; Shivram et al., 2021; Zhang and Marchisio, 2021) or Cas12a (Knott et al., 2019; Zhang H. et al., 2019; Davidson et al., 2020; Marino et al., 2020; Yu and Marchisio, 2020) by using small molecules called anti-CRISPRs. Importantly, both Cas9 and Cas12a genome editing technologies can be regulated by expression of anti-CRISPRs in *S. cerevisiae* (Li J. et al., 2018; Yu and Marchisio, 2021; Zhang and Marchisio, 2022). It will be intriguing to explore whether the use of anti-CRISPRs for Cas9 (Basgall et al., 2018; Li J. et al., 2018; Zhang and Marchisio, 2022) or Cas12a (Yu and Marchisio, 2021) might help to simultaneously reduce any potential off-target effects as well as any other unanticipated mutagenic events associated with DNA binding by Cas proteins in *S. cerevisiae*.

## Conclusion and Perspectives

Marker-free genome editing in *S. cerevisiae* typically relies on the formation and repair of Cas9-induced DNA damage to obtain specific gene deletions, insertions, or precise base substitutions. In this review article, we highlight numerous tips and tricks, which when followed, can help to ensure that genome editing experiments in *S. cerevisiae* will be successful. Importantly, we also highlight how the intended genome editing outcomes with CRISPR/Cas9 can be significantly impacted by chromatin environment. One interesting possibility to explore is whether other CRISPR technologies such as prime editing (Anzalone et al., 2020), which has largely been used in non-fungal eukaryotic systems, could efficiently generate similar editing outcomes in yeast.

Surprisingly, Cas9 (or dCas9) binding, even in the absence of DNA cleavage, can cause potentially deleterious background mutations. These mutations can occur through at least two distinct mechanisms: dCas9-induced R-loop formation inducing spontaneous cytosine deamination on the non-target strand (Laughery et al., 2019) and dCas9-induced DNA replication stress promoting the formation of larger structural variations in the genome (Doi et al., 2021). Collectively these findings are consistent with studies showing that R-loop formation can be mutagenic in living eukaryotic cells (Costantino and Koshland, 2018; Crossley et al., 2019; Gómez-González and Aguilera, 2019). Moreover, they agree with other studies showing that Cas9 (or dCas9) can inhibit other endogenous cellular processes such as double strand break repair (Clarke et al., 2018; Xu et al., 2020), replication (Wiktor et al., 2016; Whinn et al., 2019; Doi et al., 2021), and transcription (Gilbert et al., 2013; Qi et al., 2013; Clarke et al., 2018; Vigouroux et al., 2018). Along with our recent study showing that dCas9 targeting inhibits the initiation of BER *in vitro* (Antony et al., 2022), we speculate that the mechanism by which background mutations arise during genome editing may be more complex than originally anticipated. Understanding how these mutations arise in yeast or other eukaryotes can be helpful towards designing genome editing experiments that reduce or even eliminate unwanted mutations, therefore elevating the precision and accuracy of the intended edits.
